# The Phase‐Amplitude Coupling Induced by Drug Cues in Individuals With Methamphetamine Use Disorder During Withdrawal

**DOI:** 10.1002/brb3.71420

**Published:** 2026-04-16

**Authors:** Yu Tian, Yaqi Zhang, Yongxin Cheng, Juan Wang, Yuxin Ma, Yimiao Li, Jinliang Liu, Dingming Chang, Ting Xue, Kai Yuan, Gengdi Huang, Dahua Yu, Yanxue Xue, Fang Dong

**Affiliations:** ^1^ School of Digital and Intelligent Industry Inner Mongolia University of Science and Technology Baotou Inner Mongolia China; ^2^ Henan Key Laboratory of Medical Tissue Regeneration Henan Medical University Xinxiang Henan China; ^3^ School of Automation and Electrical Engineering Inner Mongolia University of Science and Technology Baotou Inner Mongolia China; ^4^ School of Science Inner Mongolia University of Science and Technology Baotou Inner Mongolia China; ^5^ Life Sciences Research Center, School of Life Science and Technology Xidian University Xi'an Shaanxi China; ^6^ Ganzhou City Key Laboratory of Mental Health The Third People's Hospital of Ganzhou City Ganzhou Jiangxi China; ^7^ Engineering Research Center of Molecular and Neuro Imaging Ministry of Education Xi'an Shaanxi China; ^8^ Xi'an Key Laboratory of Intelligent Sensing and Regulation of Trans‐Scale Life Information, School of Life Science and Technology Xidian University Xi'an Shaanxi China; ^9^ Department of Addiction Medicine Shenzhen Kangning Hospital, Shenzhen Mental Health Center Shenzhen China; ^10^ State Key Laboratory of Chemical Oncogenomics, Guangdong Provincial Key Laboratory of Chemical Genomics Peking University Shenzhen Graduate School Shenzhen China; ^11^ National Institute on Drug Dependence and Beijing Key Laboratory of Drug Dependence Peking University Beijing China; ^12^ School of Mechanical Engineering Inner Mongolia University of Science and Technology Baotou Inner Mongolia China

**Keywords:** electroencephalography, functional connectivity, methamphetamine use disorder, phase‐amplitude coupling

## Abstract

**Introduction:**

Substance use disorder (SUD) is a major global health challenge, with methamphetamine use disorder (MUD) being particularly severe due to its high addictiveness and relapse rate. The neurophysiological basis of MUD remains unclear.

**Aims and Methods:**

This study investigated cross‐frequency coupling in 46 male abstinent individuals with MUD to examine multi‐scale neural information integration. Electroencephalography (EEG) was recorded during exposure to drug‐related and neutral cues. Phase‐amplitude coupling (PAC) and phase slope index (PSI) were used to assess local and directed network connectivity, respectively.

**Results:**

Cue‐induced PAC enhancements correlated significantly with craving: left prefrontal AF3 PAC (10–14 Hz/60–82 Hz) was negatively correlated (*r* = −0.34 to −0.63, *p *< 0.05), whereas centro‐parietal CP2 PAC (6–11 Hz/74–94 Hz) was positively correlated (*r* = 0.33–0.49, *p *< 0.05). PSI analysis showed reversed prefrontal‐to‐occipital flow (*p *< 0.05) and disrupted centro‐parietal to sensorimotor connectivity (*p *< 0.05) under drug cues.

**Conclusions:**

Drug‐related cues may exacerbate craving by weakening top–down prefrontal regulation and amplifying bottom‐up sensorimotor drive. PAC and PSI represent promising cue‐reactive predictive biomarkers for evaluating the neural substrates of drug cue‐induced craving severity in individuals with MUD.

## Graphical abstract

Whole‐brain PAC and directed PSI analyses during a drug‐cue task in abstinent patients with methamphetamine use disorder revealed that drug‐related stimuli enhanced prefrontal and centro‐parietal PAC but disrupted top–down information flow from prefrontal to occipital regions. Crucially, these neural alterations were differentially correlated with subjective craving, identifying PAC and PSI as promising electrophysiological biomarkers for craving severity in addiction.

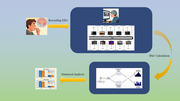

## 1 Introduction

Substance use disorder (SUD) poses a dual challenge to global mental health and judicial systems. Notably, methamphetamine use disorder (MUD) has become one of the most destructive public health crises of the 21st century due to its high addictiveness, high relapse rate, and close association with criminal behavior, and crucially, there are no FDA‐approved pharmacotherapies for the treatment of MUD or other stimulant use disorders to date. Previous research has indicated that approximately 34 million people aged 15–64 worldwide had used amphetamine‐type stimulants during the year of investigation (Zhang, Li et al. 2023). Similar to tobacco use, initial exposure to methamphetamine during adolescence significantly elevates the risk of lifelong dependence (Khan et al. 2025; Young et al. 2024; Han et al. 2021). Craving, a core symptom of MUD and a key predictor of relapse, is robustly associated with cue reactivity, thereby directly contributing to relapse behavior (Tian et al. 2024). Elucidating the temporal dynamics and neural representations of craving is critical for identifying high‐risk states and developing objective assessment tools based on electroencephalography (EEG) states or attentional bias (Paulus and Stewart 2020). Furthermore, designing interventions that target the mechanisms of craving—such as modulating the prefrontal‐striatal circuit or combining exercise training—holds promise for formulating precise strategies to reduce relapse rates (Luo et al. 2025).

Previous EEG studies on methamphetamine craving have predominantly focused on the resting state. A key finding is that a reduced frontal theta/beta power ratio in individuals with dependence accounts for over 78% of the variance in craving, establishing this metric as a sensitive indicator of craving intensity (Fadaei et al. 2025; Mori and Haruno 2022). Resting‐state microstates refer to transient, recurring patterns of brain electrical activity that reflect functional network interactions (Newson and Thiagarajan 2019). For MUD‐related craving, prior analysis of resting‐state microstates demonstrated that Microstate D—associated with the attention network—exhibited prolonged duration and heightened transition probabilities during craving, with spatial sources localized to the bilateral superior temporal gyri and parietal regions (Khajehpour et al. 2019; Gan et al. 2023). In task‐based cue‐exposure studies (fewer in number for MUD), viewing drug‐related videos elevated craving scores (from 4.6 to 6.4) in dependent individuals, which coincided with a marked decrease in Microstate C coverage—indicating that external drug cues acutely alter microstate dynamics (May et al. 2020; Liang et al. 2019). Further supporting this view, another study reported an inverse correlation between P3 amplitude and craving in dependent individuals, suggesting impaired attentional resource allocation in response to salient cues (Huang et al. 2023). Separately, research using a virtual reality EEG microstate approach has confirmed that differences in microstate transition probabilities between rest and task conditions can differentiate high‐ and low‐craving groups, providing a novel framework for ecological assessment (Lin et al. 2022; Larsen et al. 2024). Although slow‐wave prefrontal oscillations and Microstate D are well‐established resting‐state correlates of craving, it remains unclear how these microstates couple with behavior during ecologically valid task conditions. Bridging this gap is essential to understand how craving unfolds in real‐time within dynamic, compelling environments.

Cross‐frequency coupling (CFC) refers to the interaction between neural oscillations of different frequencies (Munia and Aviyente 2019). A prominent form of CFC, phase‐amplitude coupling (PAC), occurs when the phase of a low‐frequency rhythm modulates the amplitude of a high‐frequency oscillation. This mechanism is thought to orchestrate the integration of local and global information, making it a valuable metric for identifying key neural nodes associated with methamphetamine craving (Esghaei et al. 2022; Jafakesh et al. 2022). Functional connectivity (FC) quantifies the synchronization of neural activity between spatially distinct brain regions. The phase slope index (PSI) is a robust metric that determines directional coupling based on the slope of phase difference spectra (Tolomeo and Yu 2022). Its inherent resistance to volume conduction makes it particularly suitable for uncovering aberrant information flow underlying craving states (Hsu et al. 2024; Zhang, Nan et al. 2023). EEG data were collected from 46 abstinent male individuals with MUD during a drug‐cue craving task. PAC and PSI analyses were utilized to compare neural oscillations elicited by different cue conditions (Townsend et al. 2024; Zhang, Hu et al. 2025). Consequently, we hypothesized that (1) after abstinence, drug cues would trigger a significant increase in prefrontal PAC, which may signify a deficit in inhibitory control; (2) drug cues would lead to abnormal FC, compromising the coordination between the executive control and sensorimotor networks. These hypotheses address the critical research gaps that no prior work has examined PAC and PSI dynamics in individuals with MUD during drug cue exposure nor explored the link between these neural metrics and craving severity. By delineating these disturbances in local oscillatory coupling and directional information flow, this study seeks to pinpoint novel electrophysiological targets for therapeutic intervention (Jafakesh et al. 2022; Ceceli et al. 2023; Samiee and Baillet 2017).

## 2 Materials and Methods

### 2.1 Subject Screening

This study was approved by the Peking University Institutional Review Board. Written informed consent was obtained from all participants prior to the commencement of the study. The study cohort consisted of 46 right‐handed, Han Chinese males diagnosed with MUD, with recruitment restricted to male participants due to practical constraints that the study was conducted in a male‐only compulsory drug rehabilitation center in Shenzhen, where female individuals with MUD under compulsory abstinence were not available during the study period. All participants were ascertained during a period of enforced abstinence from this detoxification facility in Shenzhen, China. Owing to the recruitment setting of a male compulsory drug rehabilitation center, this investigation was restricted to male individuals with MUD, who had a mean age of 36.4 ± 8.1 years. The inclusion criteria comprised the following: (1) fulfillment of the DSM‐V diagnostic criteria for MUD (Hasin et al. 2013); (2) normal visual and auditory acuity; (3) a minimum 2‐week withdrawal period, absent of significant symptoms (e.g., drowsiness and restlessness); (4) no comorbid psychotic disorders; (5) the absence of major systemic illnesses (e.g., cardiac and hepatic); and (6) provision of written informed consent by all participants. All participants had undergone 1–2 years of methamphetamine withdrawal and were medication‐free prior to the experiment (no psychotropic drugs, detoxification medications, or other relevant medications taken within the study period). Subjects were excluded according to the following criteria: (1) a history of illicit substance use other than methamphetamine (e.g., heroin); (2) moderate‐to‐severe psychiatric disorders, including a lifetime history of psychosis or a current diagnosis of post‐traumatic stress disorder, major depressive disorder, panic disorder, or other anxiety disorders; (3) neurological conditions that could interfere with study procedures, such as epilepsy or Parkinson's disease; (4) a history of head trauma leading to loss of consciousness exceeding 30 min; and (5) the presence of cognitive impairment. Demographic characteristics of the enrolled participants are summarized in Table 1. As the EEG recordings were obtained from a single cue‐elicited task that was divided into drug‐cue and neutral‐cue conditions, data from both conditions were derived from the same set of participants. This approach ensured that there were no significant differences in key demographic variables, including age, height, weight, and education level. Following the assessment of craving levels via the MUD Severity Scale, participants underwent a standardized scalp cleansing procedure to facilitate the collection of high‐fidelity EEG data.

**TABLE 1:** Demographic characteristics of the participants.

Demographic variables	Individuals with MUD (*N *= 46)
Sex (male), *N* (%) Age range (years) Age (years), mean ± SD Education years (years), mean ± SD Age at MA onset (years), mean ± SD MA‐use duration (years), mean ± SD Dose per episode (g), mean ± SD Days since last abstinence (days), mean ± SD Craving score, mean ± SD DSM‐5 score, mean ± SD	46 (100) 26–51 36.4 ± 8.1 7.5 ± 3.7 23.5 ± 9.1 9.4 ± 5.4 0.4 ± 0.35 628.9 ± 200.7 5.3 ± 2.1 5.6 ± 2.0

Abbreviation: MUD, methamphetamine use disorder.

### 2.2 Experimental Design and Procedures

**FIGURE 1:**
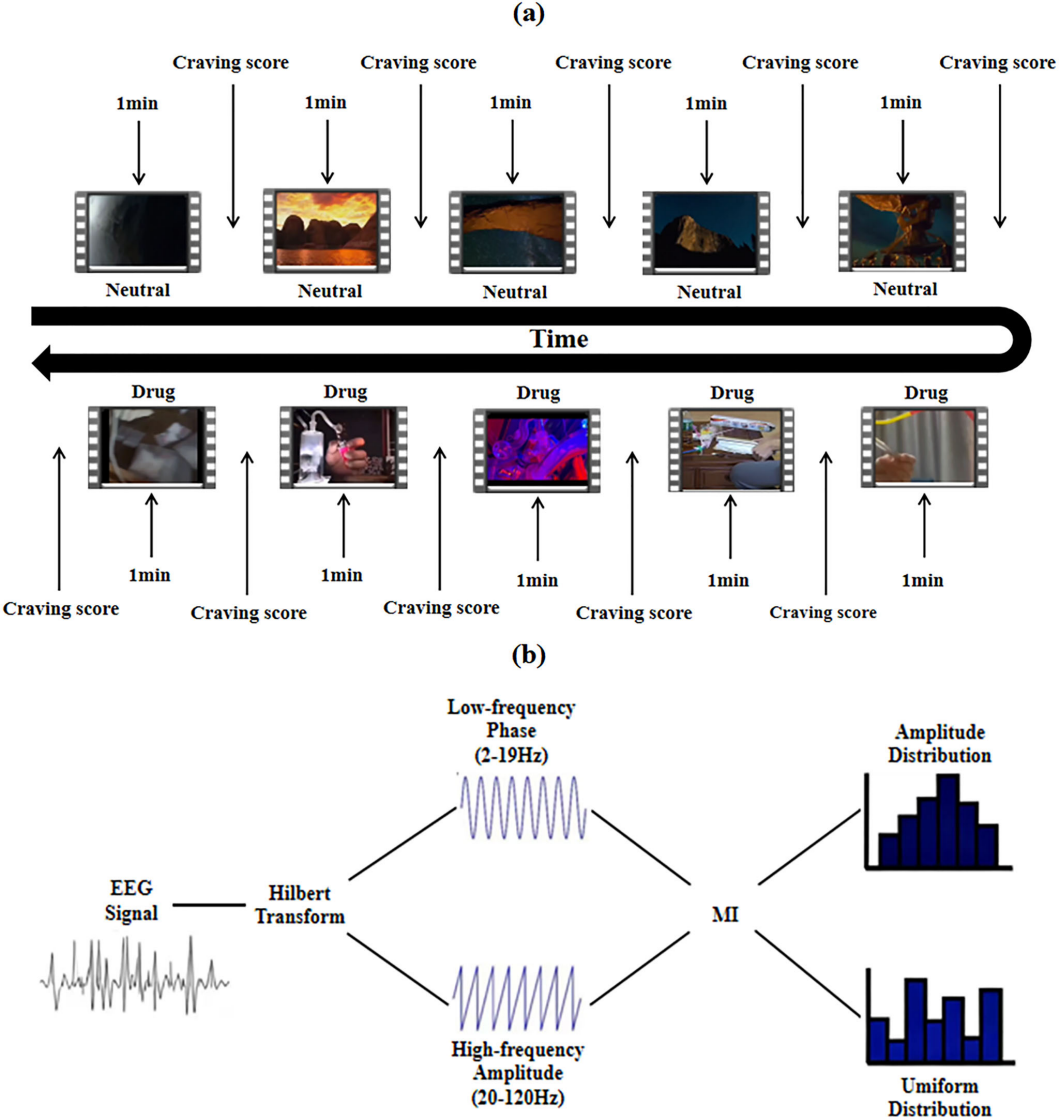
Experimental design and computational procedure for PAC: (a) cue‐task experimental paradigm flow; (b) PAC calculation flowchart. The flowchart shows the PAC calculation process in detail. EEG, electroencephalography; MI, modulation index.

The experimental paradigm was implemented in E‐Prime 3.0 (Psychology Software Tools, Inc., Sharpsburg, PA, USA). The stimulus set consisted of five methamphetamine‐related and five neutral video clips, which were carefully selected for the study, with no repetition among the five videos within each stimulus category. The cue‐induced craving paradigm comprised two categories of visual stimuli: methamphetamine‐related videos, which showcased the substance, smoking procedures, exhaled vapors, and paraphernalia assembly; and neutral videos, which concurrently presented scenes of natural scenery such as grasslands, hills, and lakes. The video sets were parallel in all aspects except for content (methamphetamine‐related vs. natural scenery). Following a standardized protocol, the five neutral cue videos were presented sequentially first, followed by the five drug‐related cue videos (the two types of cue videos were not presented alternately). A variable interstimulus interval of 10–15 s separated the offset of one video from the onset of the next, serving to demarcate trials and allow participants to report their craving levels (Figure 1a).

Pre‐experimental scales were administered to all participants. A 64‐electrode EEG cap was fitted to the scalp, with supplementary electrodes placed on the arms and chest. Stable electrode‐scalp impedance was ensured through the application of conductive gel, maintaining levels below 10 kΩ. The experimental session then proceeded for approximately 15 min, culminating in the removal of the cap. No baseline craving data were collected prior to cue exposure.

### 2.3 Data Acquisition and Preprocessing

EEG signals were recorded with the NeuSen W wireless digital EEG acquisition system (a domestically developed system from Neuracle, Inc., Shanghai, China). This system integrates high‐performance flexible wet electrodes into a lightweight headset, with the montage following the International 10–20 system and a built‐in reference electrode for real‐time referencing. Boasting a configurable sampling rate of up to 1000 Hz, the system employs active impedance monitoring with real‐time tracking to automatically keep electrode‐scalp impedance under 10 kΩ, thereby guaranteeing optimal signal quality. All multimodal data are acquired in synchrony and recorded via an uninterrupted digital stream to the dedicated NeuroHub platform.

Offline analysis of the acquired EEG data was performed using MATLAB R2024b, following a standardized processing pipeline: (1) electrode localization and removal of superfluous electrodes: electrodes were positioned according to the International 10–20 System; 59 EEG electrodes were retained for analysis, whereas non‐EEG electrodes (HD‐EMG, ECG, EDA/GSR, EOG) were excluded for recording irrelevant physiological signals with no analytical significance for the study's core objectives; (2) filtering: a high‐pass filter with a cutoff frequency of 0.1 Hz and a low‐pass filter with a cutoff frequency of 120 Hz were applied to eliminate nonphysiological signals, and notch filters at 50 Hz and 100 Hz were used to suppress industrial frequency interference; (3) electrode re‐referencing: with the FCz electrode, the original online reference, incorporated into the array, a whole‐brain average reference was applied, which mitigates interference from electrocardiographic artifacts and interhemispheric potential differences by utilizing the average potential of all valid recording channels; (4) interpolation of bad electrodes: Electrodes that failed to accurately capture EEG signals (designated as bad electrodes) were excluded and replaced via interpolation from adjacent electrodes to enhance data quality; (5) rejection of artifact‐laden epochs: signal segments contaminated by artifacts arising from activities such as swallowing saliva and body movements were discarded to facilitate clearer analysis; (6) independent component analysis (ICA): ICA was employed to remove artifacts associated with eye movements and blinks, thereby preserving genuine signals to the greatest extent possible.

### 2.4 PAC Calculation

For PAC analysis, EEG signals were parsed into phase‐ and amplitude‐defined components. The low‐frequency phase band (2–19 Hz) covered delta, theta, alpha, and beta rhythms, whereas the high‐frequency amplitude band (20–120 Hz) captured gamma activity. The low‐frequency phase and high‐frequency amplitude were derived by applying the Hilbert transform. To quantify PAC, we computed the modulation index (MI) chosen for its robust resistance to spurious coupling and high sensitivity for genuine PAC in EEG signals and employed cluster‐based permutation tests to identify electrodes and frequency bands with statistically significant between‐group differences in PAC strength (Figure 1b). A normalization procedure was applied to the PAC metric to improve robustness and mitigate parasitic coupling (Tort et al. 2010). Specifically, PAC was computed over successive 3‐s epochs, and the epoch‐averaged value was utilized as the definitive metric (Aru et al. 2015). These analyses were performed employing the Tensorpac toolbox (Combrisson et al. 2020).

### 2.5 FC Calculation

We employed the PSI to estimate directionality in long‐range connectivity during the drug‐cue induction. This metric derives the direction of information transfer from the stability of phase slope relationships across trials, providing a robust measure of inter‐regional causal relationships that is less susceptible to volume conduction. A constant time lag between signals corresponds to a linear phase shift across frequencies (Nolte et al. 2008). On the basis of this principle, the PSI serves as a robust metric for estimating directional influence. To mitigate edge artifacts, a relatively long segment of 3000 ms was employed following the application of a Hanning window and extraction of Fourier coefficients. Building on the preceding PAC findings, directed connectivity was assessed via the PSI. PSI was computed between the AF3 electrode and all other 58 electrodes under both drug‐cue and neutral conditions, within the frequency ranges of 10–14 and 60–82 Hz, respectively. A corresponding analysis was performed for the CP2 electrode, evaluating its connectivity with the other 58 electrodes in the 6–11 and 74–94 Hz bands. A significantly positive PSI value denotes a predominant information outflow from the AF3/CP2 electrodes to others. Conversely, a negative value indicates a reversal of this flow, signifying information inflow. A value approaching zero implies negligible directional information transfer.

### 2.6 Statistical Analysis

Statistical analyses were performed using paired *t*‐tests and Pearson's correlation. To correct for multiple comparisons and identify statistically significant differences in PAC, a cluster‐based permutation test was implemented in MNE‐Python, with the significance level set at *p* < 0.05 (Maris and Oostenveld 2007). To quantify the linear relationship between PAC values and craving scores, we computed Pearson's correlation coefficients. For the within‐frequency PSI analysis, PSI values were averaged across electrode pairs. We then employed paired‐sample *t*‐tests to assess the directional differences in these averaged PSI values between the neutral and drug‐cue task conditions. Finally, paired‐sample *t*‐tests were also applied to evaluate the differences in subjective craving scores across the two conditions.

## 3 Results

### 3.1 Cue‐Induced Craving Elevation

**FIGURE 2:**
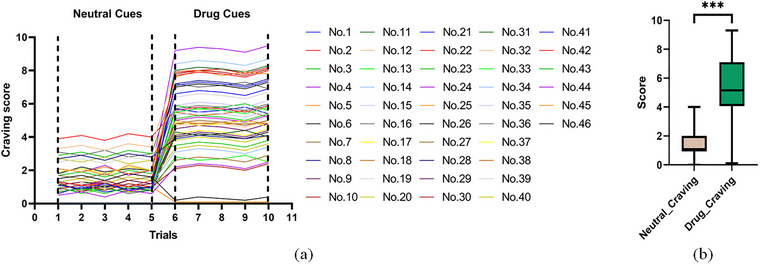
Individual craving trajectories and group differences in individuals with MUD. (a) Spaghetti plot showing raw craving scores of each participant across all trial points; Trial 1–5 = neutral cues; and Trial 6–10 = drug cues. Craving remained stable at a plateau without obvious habituation across repeated trials. (b) Group comparison of averaged craving scores between neutral and drug conditions. (ns, not significant. *p < 0.05; **p < 0.01; ***p < 0.001.)

To assess the psychological response to drug cue exposure in individuals with MUD, we first visualized individual participant trajectories of craving ratings across all trials using a spaghetti plot (Figure 2a). This figure displays raw craving scores at each time point for every participant, allowing inspection of the full distribution of responses and confirming that observed effects are not driven by outliers. Critically, following the onset of methamphetamine‐related cues, craving ratings entered a sustained plateau across all subsequent trials, with no visible downward trend indicative of response habituation. Building on this stable pattern, we then compared mean craving scores following methamphetamine‐related and neutral cue presentation (Figure 2b). As expected, descriptive statistics showed that craving scores were significantly higher after drug cue exposure than neutral cue exposure. A paired‐samples *t*‐test verified this significant difference with a large effect size, confirming that methamphetamine‐related cues robustly and specifically elicit heightened drug craving in individuals with MUD. To maintain methodological consistency with the preprocessing of PAC and PSI data (where we averaged across five trials for each cue type), craving scores were averaged for each cue type per participant before statistical comparison. The visually stable, non‐declining pattern of craving responses across repeated drug cue presentations, as shown in Figure 2a, supports the validity of this averaging strategy.

### 3.2 Abnormal PAC Changes

**FIGURE 3:**
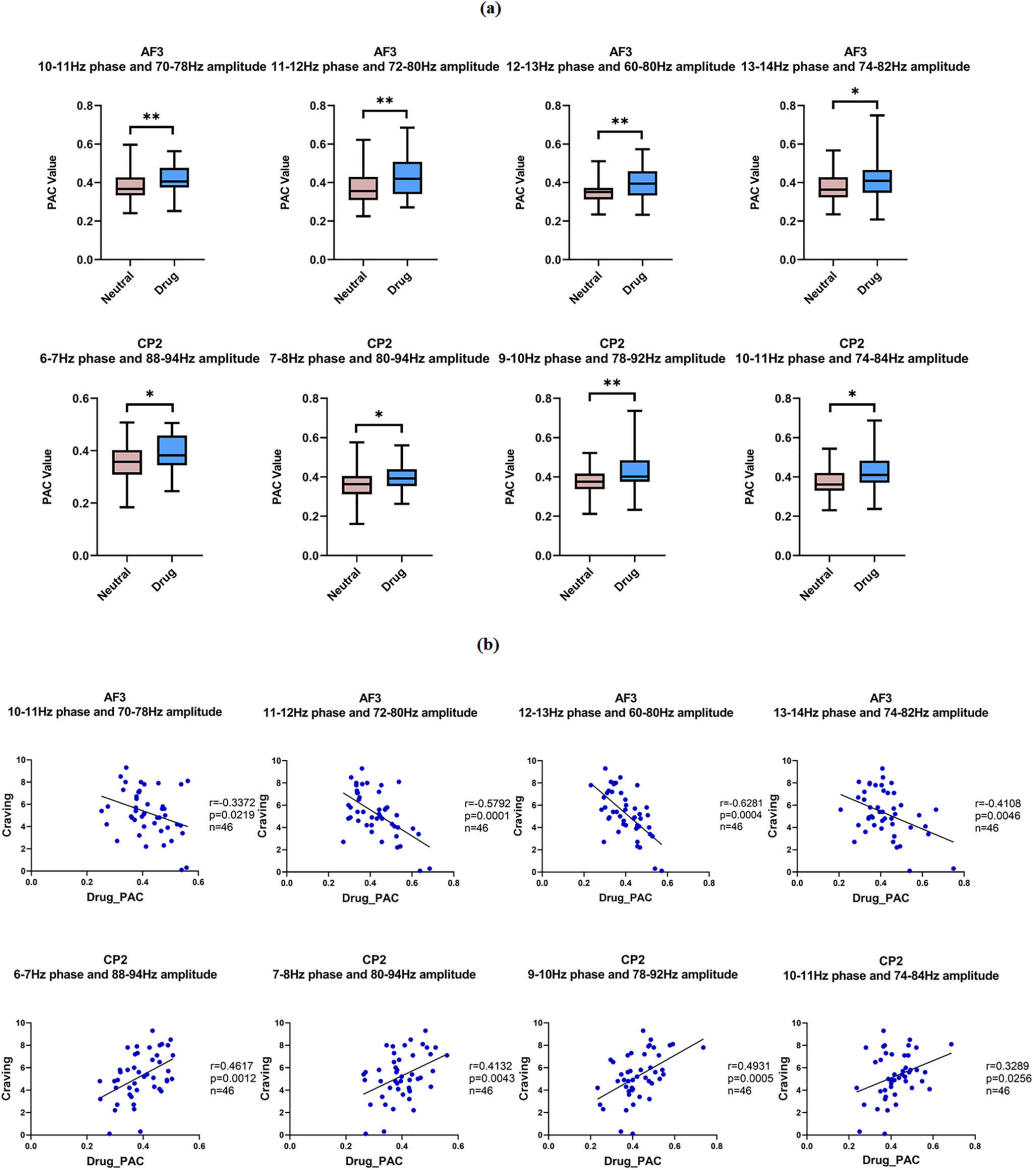
Differences in PAC in individuals with MUD after drug cue reactivity: (a) differences in PAC values between neutral and drug (ns, not significant; *p < 0.05; **p < 0.01); (b) correlation between drug_PAC and craving. PAC, phase‐amplitude coupling.

Assessment of electrophysiological activity in abstinent individuals with MUD revealed a specific enhancement in PAC at the AF3 electrode, triggered by drug‐related cues relative to neutral videos. To quantify the effect within significant clusters, the time‐course data from each cluster were averaged for every subject and subsequently subjected to paired‐samples *t*‐tests. The results demonstrated a significant increase in both the 10–14 Hz phase synchronization and the 60–82 Hz amplitude oscillations (Figure 3a). Similarly, during the drug‐cue task, a significant increase in PAC was observed at electrode CP2, modulated by the theta rhythm at 6–11 Hz and coupled with high‐gamma amplitude at 74–94 Hz (Figure 3a). Notably, PAC was significantly enhanced in the left prefrontal region, along with a frequency‐specific increase in the centro‐parietal cortex, in individuals with MUD during the experimental task.

A significant negative correlation was found between average craving scores across the five drug‐cue videos and the drug cue‐induced PAC value recorded from the AF3 electrode. This value represented the modulation of high‐gamma amplitude (60–82 Hz) by the phase of concurrent theta/low‐alpha oscillations (10–14 Hz), which was elicited during drug‐cue video exposure (Figure 3b). Conversely, a significant positive correlation was observed between average craving scores across the five drug‐cue videos and the drug cue‐induced PAC values at electrode CP2, which coupled the theta‐alpha (6–11 Hz) phase with high‐gamma (74–94 Hz) amplitude during drug‐cue video exposure (Figure 3b).

### 3.3 Abnormal FC Changes

**FIGURE 4:**
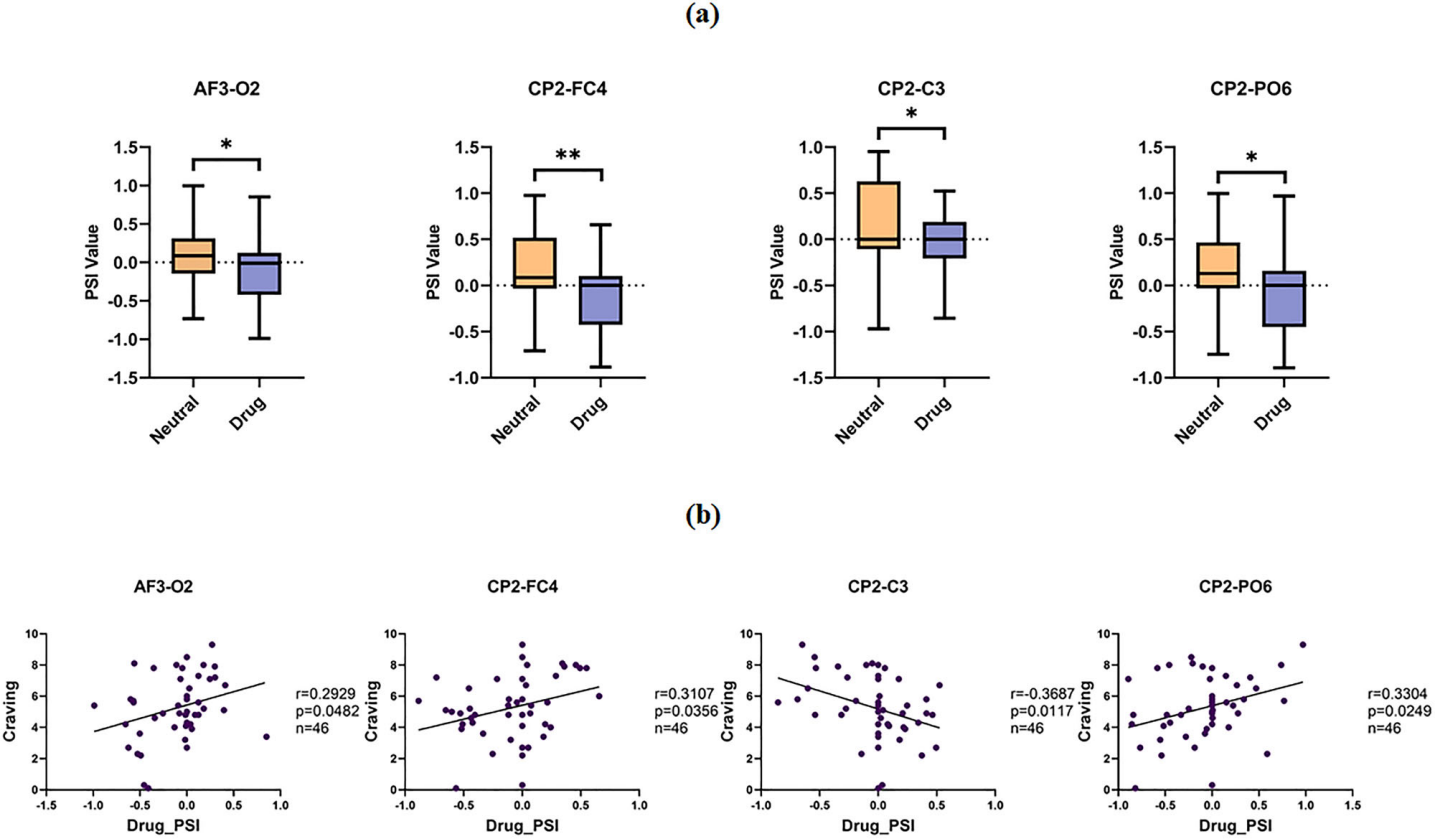
Differences in PAC in individuals with MUD after drug cue reactivity: (a) differences in PSI values between neutral and drug (ns, not significant; *p < 0.05; **p < 0.01); (b) correlation between drug_PSI and craving. PSI, phase slope index.

The reduction in PSI values and reversed information flow from neutral to drug cue conditions were consistent across all 46 MUD participants, with only minor individual differences in the reduction magnitude of a small number of subjects. Positive PSI values observed between the AF3 and O2 electrodes in abstinent individuals with MUD during neutral video viewing provide electrophysiological evidence for a directed fronto‐occipital information transfer. However, the presentation of drug‐related cues led to a significant reduction in PSI between the AF3 and O2 electrodes, which fell to near‐zero levels. This demonstrates that the information flow was predominantly from O2 to AF3, thereby implicating a specific pathway during cue processing (Figure 4a). Similarly, for the CP2 electrode, a positive PSI was observed in its connections with FC4, C3, and PO6 during neutral video viewing, indicating a flow of information emanating from CP2 to these electrodes. During the presentation of drug‐cue videos, a marked attenuation of the PSI was observed between the CP2 electrode and the FC4, C3, and PO6 electrodes, with values approaching zero. This pattern signifies a directed flow of neural information from FC4, C3, and PO6 toward the CP2 electrode, identifying CP2 as the informational sink (Figure 4a).

Of the electrode pairs analyzed, a significant positive correlation with craving scores was observed in the PSI values of the AF3‐O2 derivation, localized specifically to the AF3 electrode (Figure 4b). Analysis of the CP2 electrode similarly revealed that FC for the CP2‐FC4 and CP2‐PO6 pairs was positively correlated with craving scores. In contrast, the CP2–C3 pair exhibited a negative correlation (Figure 4b).

## 4 Discussion

This study provides the first evidence linking alterations in PAC to self‐reported craving in abstinent individuals with MUD during drug‐cue exposure. Our findings revealed that exposure to drug cues, compared to neutral videos, induced a significant enhancement of PAC in the left prefrontal cortex, characterized by phase frequencies in the 10–14 Hz range modulating amplitude frequencies between 60 and 82 Hz. Concurrently, we observed a significant reduction in the PSI between electrodes AF3 and O2. Furthermore, drug cue‐induced PAC values demonstrated a significant negative correlation with craving scores, whereas the corresponding PSI measures showed a significant positive correlation. Collectively, these results suggest that the enhanced coupling in the left prefrontal cortex may be implicated in a compromised inhibitory function of the executive control network, potentially associated with a reverse recruitment of prefrontal resources by the visual‐salience network. Consistent with this, drug‐cue videos specifically potentiated PAC in the centro‐parietal cortex, characterized by 6–11 Hz phase modulating 74–94 Hz amplitude. This was accompanied by a widespread decrease in PSI from the CP2 electrode. We found compellingly link between these cue‐induced PAC/PSI modulations and craving scores, which were predominantly positive, with a notable negative correlation observed for PSI with C3. Collectively, these findings point to a model where craving is associated with the dysfunctional hyper‐capture of the sensory‐motor network by salient drug cues, resulting in an overload of passive input to the central multisensory integration hub.

Emerging evidence suggests that PAC serves as a novel biomarker, providing critical insights into the synchronization of neural oscillations across frequencies. Consequently, PAC has garnered significant research interest in a range of neurological and psychiatric disorders, including Alzheimer's disease, ADHD, autism spectrum disorder, obsessive‐compulsive disorder, depression, and schizophrenia. Wongveerakul et al. demonstrated that acute methamphetamine exposure elicits immediate neural sensitization of the reward system by reducing theta power and enhancing gamma oscillations in the nucleus accumbens while concurrently preserving theta–gamma PAC. This finding provides key evidence for understanding the early neural mechanisms of addiction (Wongveerakul et al. 2024). Additionally, Ye et al. reported that prolonged (10‐h), low‐dose ketamine exposure potentiates delta‐high frequency CFC in the dorsal striatum, proposing the reinforcement of corticostriatal PAC synchronization as a key underlying mechanism for the induced neuroplasticity (Ye et al. 2018). Our results indicate that drug cue exposure triggers distinct PAC and FC patterns in the left prefrontal and centro‐parietal cortices. This suggests that methamphetamine‐induced neuroadaptations alter neural dynamics, thereby linking enhanced craving to impaired inhibitory control. This supports the prevailing view that MUD severely compromises cognitive control and sensory integration, reflected in significant shifts in neuro‐oscillatory coupling. Caixeta et al. demonstrated that in awake rats, ketamine—an NMDAR antagonist—enhances theta‐HFO coupling in the hippocampus in a dose‐dependent manner. Conversely, its impact on theta–gamma PAC is biphasic, with enhancement at low doses versus disruption at higher doses. This dissociation indicates that the breakdown of theta–gamma PAC may be a critical electrophysiological correlate of the cognitive deficits seen in ketamine models of schizophrenia (Caixeta et al. 2013). In a similar vein, Blain‐Moraes et al. noted that sevoflurane‐induced unconsciousness does not involve the anteriorization of prefrontal alpha power. Rather, it manifests as a significant disruption in parietal theta‐alpha PAC, coupled with a reversible reduction in whole‐brain alpha‐phase synchrony. These findings imply that alterations in consciousness may be more intimately tied to the impairment of phase‐coordinated long‐range neural communication (Blain‐Moraes et al. 2015). Aligning with previous evidence, abstinent individuals with MUD displayed a significant enhancement of PAC at the left prefrontal AF3 electrode (10–14 phase, 60–82 Hz amplitude) upon drug‐cue exposure. A concomitant reduction in FC was observed between AF3 and the occipital O2 electrode, pointing to a dysfunctional interplay between the executive and sensory processing networks. This finding suggests that the heightened PAC may signify a neural effort to mobilize top–down control mechanisms to counteract drug craving (Parrilla‐Carrero et al. 2018). Furthermore, drug‐cue exposure was found to enhance PAC between the 6–11 Hz phase frequency and the 74–94 Hz amplitude frequency at the central‐parietal CP2 electrode. Concurrently, a significant reduction in FC was observed between CP2 and other electrodes. Collectively, these results suggest that the enhanced PAC in the central‐parietal region does not reflect a compensatory mechanism. Instead, it may indicate an automated intensification of sensory‐motor integration in response to drug‐related cues during a state of high craving.

Further analysis identified a compelling link between neural dynamics and subjective experience in individuals with MUD. A significant negative correlation emerged at the left prefrontal AF3 electrode, where the PAC value (10–14 Hz phase/60–82 Hz amplitude coupling) during drug‐cue exposure diminished with increasing craving scores. This suggests that intense craving may undermine a key prefrontal mechanism—specifically, the coupling of alpha/low‐beta phase to high‐gamma amplitude that potentially underlies inhibitory control—thereby reflecting a compromised prefrontal function (Dakhili et al. 2022; Yang et al. 2025). Conversely, PAC at the CP2 electrode (6–11 Hz phase to 74–94 Hz amplitude) exhibited a positive correlation with craving. This indicates that intense craving potentiates automated processing in parietal sensorimotor networks, whereas the weakened coupling during low craving signifies a reallocation of cognitive resources. To assess the functional significance of the PAC results, a directional PSI analysis was conducted. It identified impaired FC from the occipital O2 electrode to the left prefrontal AF3 electrode under drug‐cue exposure. This reversed information flow signifies a shift from top–down cognitive control to a bottom‐up stimulus‐driven paradigm, where visual hyperactivation overrides prefrontal regulation, resulting in disinhibition (Zhang, Yu et al. 2025). Meanwhile, the prefrontal cortex exhibited heightened local gamma activity despite impaired global neural synchronization, indicating a state of maladaptive excitation that fails to suppress impulses. This pattern underscores a mechanism in which craving behaviors are governed by a disinhibited and habit‐driven system. A significantly reduced PSI value at the CP2 electrode, indicating unidirectional information inflow, points to a compromised ability for temporal coordination and cross‐regional synchronization. We propose that this reflects a capture of the sensorimotor cortex by the craving‐related salience network, converting parietal activity into a passive state and disabling its active integration (Tian et al. 2024; Ekhtiari et al. 2022). The study reveals that during craving, the central‐parietal circuitry is transformed into a passive hub for drug cues. This dysfunction amplifies craving through a critical mechanism: the breakdown of inhibitory control and the concurrent intensification of sensorimotor automation.

A residual “drug signature” persists in clustered neurons of abstinent individuals with MUD. When exposed to drug cues, these networks exhibit a robust, task‐locked spike in theta/alpha/low beta‐to‐gamma PAC, concurrently engaging the left prefrontal and centro‐parietal cortices. This PAC enhancement was strictly confined to the cue segments, demonstrating a direct and specific response to the salient stimuli (Zhang, Yu et al. 2025). A striking dissociation was observed between prefrontal and parietal sites. Elevated PAC at AF3 correlated with reduced craving, suggesting a compensatory override of prefrontal inhibitory systems. In contrast, increased PAC at CP2 was associated with intensified craving, positioning the parietal lobe as a potential “gain amplifier” for craving drive. The directional PSI results further demonstrated that drug cues trigger a unidirectional boost in bottom‐up signaling, effectively reversing the executive network's top–down regulation and overloading the sensory‐motor network. This process is marked by a persistent decoupling and unidirectional capture, offering a quantifiable electrophysiological marker of MUD. Future studies combining multimodal methodologies could target this abnormal CFC, potentially guiding novel cognitive therapeutic strategies for withdrawal.

## 5 Limitations

This study has several limitations. Notably, the data were sourced solely from a male‐only compulsory rehabilitation facility in Shenzhen, where female MUD patients were not available for recruitment, resulting in the exclusive inclusion of male participants. Consequently, the findings may not be generalizable to female populations, and future studies should recruit a mixed‐gender cohort to verify and expand our results, which remains an important area for further investigation. Furthermore, this study did not perform source signal analysis, limiting the spatial localization of both PAC and FC to the sensor level. Additionally, the cross‐sectional design precludes causal inferences regarding the relationships among patterns of substance use, abstinence duration, and neurophysiological indices. To further elucidate the addiction mechanisms, subsequent studies will leverage an expanded mixed‐gender cohort, refined source reconstruction algorithms, and longitudinal follow‐ups. Combined with assessments of pharmacological interventions, this multifaceted approach will systematically map the dynamic effects of methamphetamine on brain plasticity in individuals with MUD. Notably, no baseline craving data were collected, and the study was not preregistered—correlation analyses should thus be considered exploratory.

## Author Contributions

Yaqi Zhang: validation, investigation, resources. Juan Wang: formal analysis, data curation. Yuxin Ma: methodology. Yu Tian: methodology, software, formal analysis, investigation, writing – original draft. Yimiao Li: conceptualization. Yongxin Cheng: conceptualization, methodology. Dingming Chang: software, visualization. Jinliang Liu: investigation, data curation. Dahua Yu: writing – review and editing, resources, funding acquisition. Fang Dong: writing – review and editing.

## Funding

This work was supported by the National Natural Science Foundation of China (32161143022); STI2030‐Major Projects (2022ZD0214500); Chinese National Programs for Brain Science and Brain‐like Intelligence Technology (2022ZD0214500); National Natural Science Foundation of China (82260359, 82371500, U22A20303, 61971451); Natural Science Foundation of Inner Mongolia (2023QN08007, 2025MS08027, 2025MS08098); and the Development Program for Young Talents of Science and Technology in Universities of Inner Mongolia (NJYT24030). This work was partially supported by the Fundamental Research Funds for the Universities of Inner Mongolia (2023QNJS204, 2023QNJS206, 2024QNJS119).

## Ethics Statement

The study protocol was reviewed and approved by the Peking University Institutional Review Board (PUIRB Approval No. 2025044‐Re01). All procedures were conducted in accordance with the Declaration of Helsinki. Written informed consent was obtained from every participant after the procedures, risks, and rights had been fully explained.

## Conflicts of Interest

The authors declare no conflicts of interest.

## Data Availability

The dataset used in this study is available from the corresponding author upon reasonable request.
